# Supplementary material for: Synthesis and Self-Assembly of Multistimulus-Responsive Azobenzene-Containing Diblock Copolymer through RAFT Polymerization
Source: Polymers (Basel). 2019 Dec 6;11(12):2028. doi: 10.3390/polym11122028 (PMC6960709; doi:10.3390/polym11122028)
Supplement: Supplementary file 1 [file polymers-11-02028-s001.pdf]

Supplementary materials

Synthesis and Self-Assembly of Multistimulus-Responsive Azobenzene-Containing Diblock Copolymer through RAFT Polymerization

Po-Chih Yang*, Yueh-Han Chien, Shih-Hsuan Tseng, Chia-Chung Lin and Kai-Yu Huang

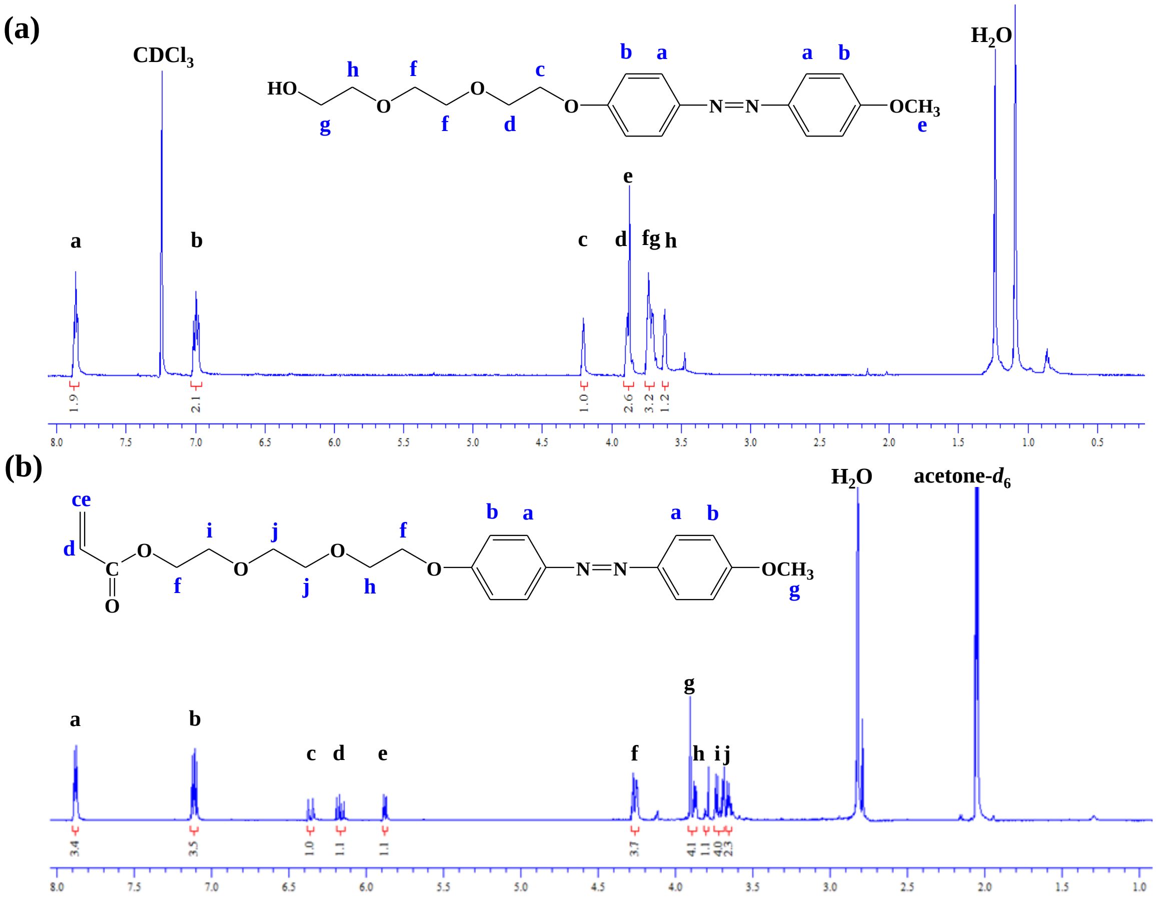

Figure S1. ^{1}H NMR spectra of azobenzene compounds (**a**) 1 and (**b**) 2.

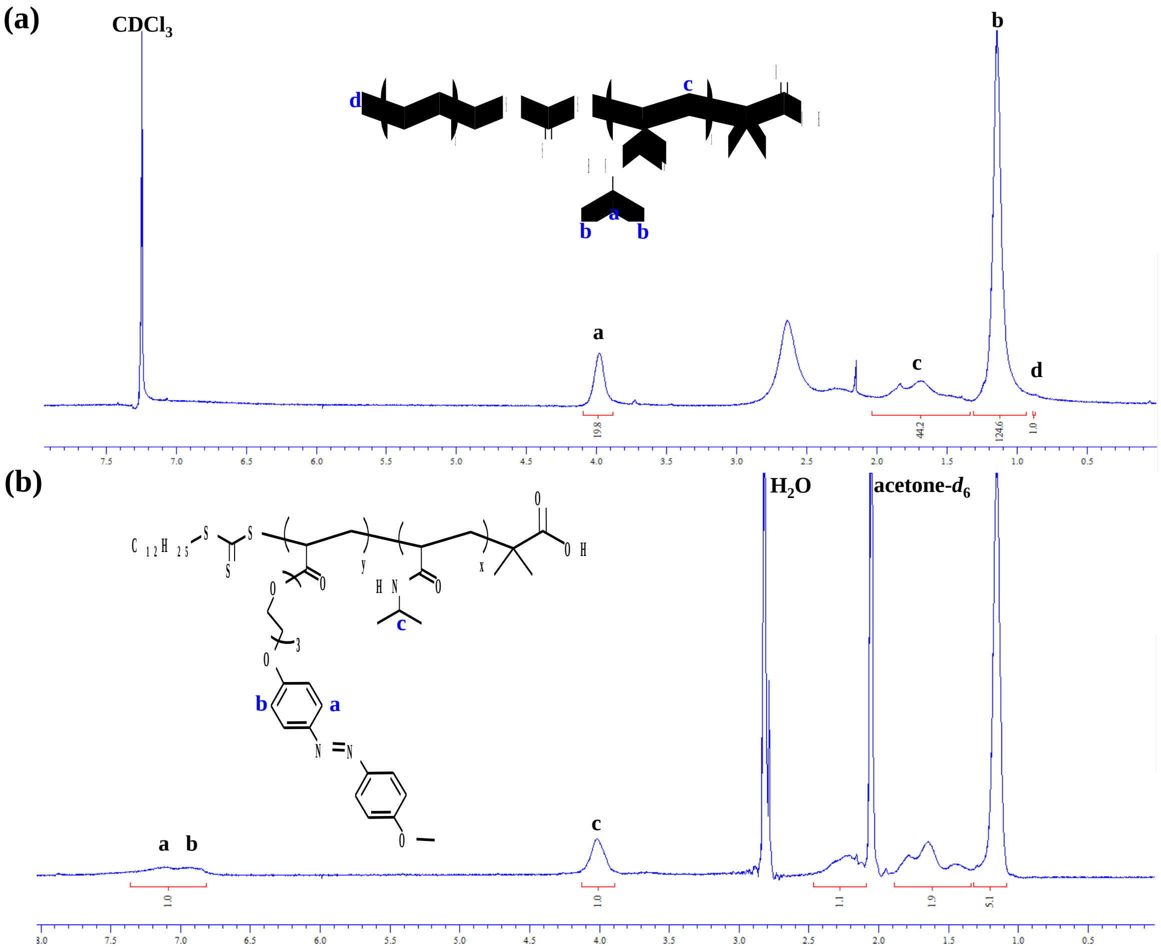

Figure S2. ^{1}H NMR spectra of polymers (**a**) NIPAM macro-CTA and (**b**) poly(NIPAM-*b*-Azo).

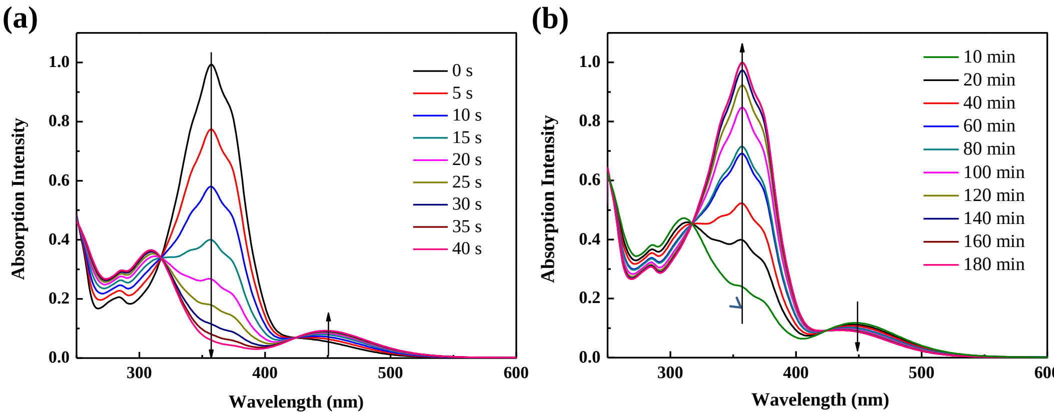

Figure S3. (**a**) UV-vis spectra of Azo monomer under various irradiation durations and (**b**) stability of the UV-vis spectra of Azo monomer in the dark.

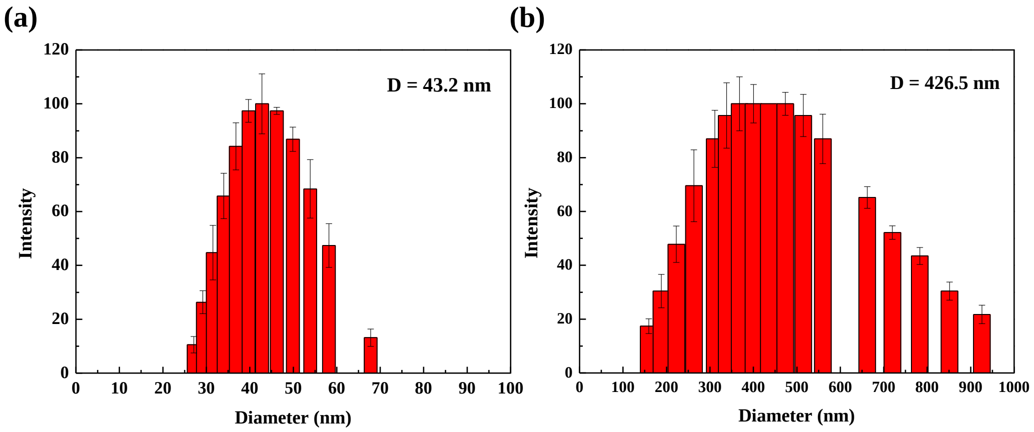

Figure S4. Particle size distributions of poly(NIPAM) at (**a**) 25 and (**b**) 40 °C.

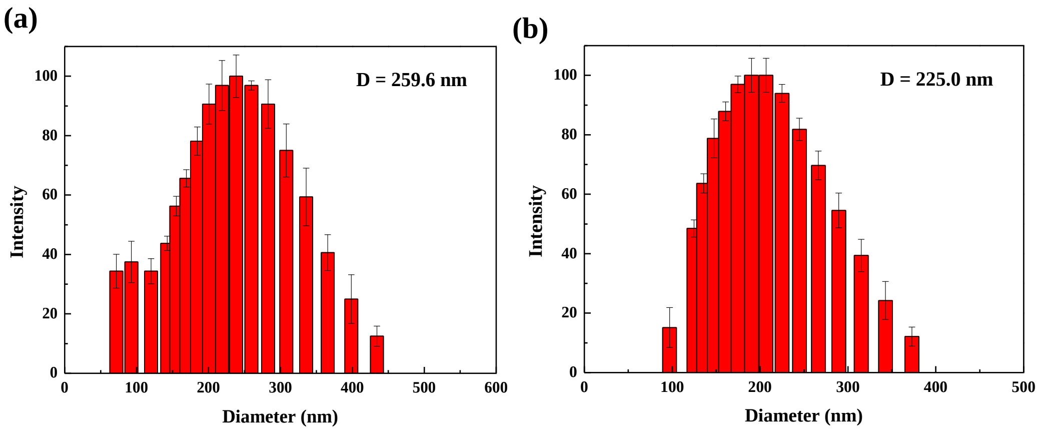

Figure S5. Particle size distributions of poly(NIPAM-*b*-Azo) after chelation of (**a**) Ba^{2+} and (**b**) Ru^{3+}. Metal ion concentration in water: 1.0×10^{-4} M.
